# Gastrointestinal Endoscopy in the Era of COVID-19

**DOI:** 10.3389/fmed.2020.587602

**Published:** 2020-11-26

**Authors:** Abhilash Perisetti, Hemant Goyal, Neil Sharma

**Affiliations:** ^1^Department of Gastroenterology and Hepatology, University of Arkansas for Medical Sciences, Little Rock, AR, United States; ^2^The Wright Center for Graduate Medical Education, Scranton, PA, United States; ^3^Mercer University School of Medicine, Macon, GA, United States; ^4^Division of Interventional Oncology & Surgical Endoscopy (IOSE), Parkview Cancer Institute, Fort Wayne, IN, United States; ^5^Indiana University School of Medicine, Fort Wayne, IN, United States

**Keywords:** coronavirus, coronavirus (2019-nCoV), SARS-CoV-2 infection, pandemic (COVID-19), endoscopy, gastrointestinal disease, fellowship and training

## Abstract

Coronavirus disease 2019 (COVID-19) is caused by severe acute respiratory syndrome coronavirus-2 (SARS-CoV-2), which led to a worldwide pandemic that started in early 2020. Healthcare systems across the world encountered an unprecedented surge of COVID-19 patients resulting in more than half a million deaths globally. COVID-19 has affected multiple sub-specialties and procedure-related fields, including gastroenterology. Gastrointestinal (GI) endoscopy centers are specialized units where thousands of endoscopies are performed annually. A significant proportion of these procedures are affected due to the national and regional lockdowns across the globe. To adapt to this rapidly evolving situation, endoscopy centers have undergone significant changes and have taken unprecedented precautions to avoid the transmission of the virus. However, endoscopy centers are going through financial strain due to a reduction in the number of procedures from lockdowns and fear of virus transmission. Theoretically, endoscopies could add to the disease transmission as SARS-CoV-2 has shown to be present in the GI secretions. Multiple precautions such as mandatory use of face masks, safe distancing, use of barriers between the endoscopists and patients, negative pressure rooms, extended use of personal protective equipment, and volume reduction have been taken to decrease the risk of disease transmission by these centers. Moreover, pre-endoscopy COVID-19 testing has now become the norm. In this review, we highlight the significant changes assumed by the endoscopy center. Furthermore, we discuss cost-related concerns of pre-endoscopy COVID-19 testing, the downtime and delays related to the procedures, and effects of rescheduling. As the pandemic progresses through multiple phases, endoscopy centers should use a dynamic approach to adapt and strive to provide the best patient care.

## Introduction

Coronavirus disease 2019 (COVID-19) is caused by severe acute respiratory syndrome coronavirus-2 (SARS-CoV-2). Within a few months, it has led to a pandemic of unprecedented levels affecting multiple countries with >16 million cases and >650,000 deaths as of July 26, 2020 (1). The pandemic has caused duress for medical systems and hospitals worldwide. Initially, it was believed that respiratory manifestations dominate the presentation of COVID-19. As the experience with the pandemic evolved, extrapulmonary manifestations are increasingly being recognized (2–6). The Centers for Disease Control and Prevention (CDC) added multiple symptoms as a part of the COVID-19 presentation, which also includes gastrointestinal (GI) manifestations such as nausea, vomiting, dysgeusia, pancreatitis, hepatitis, colitis, etc. (6–10). Additionally, the virus has also been shown to be present in GI secretions. Specialties such as gastroenterology and surgery have been directly affected by COVID-19. This pandemic has had a disruptive effect on the workflow and safety of endoscopists, ancillary staff, and patients. Shortages of personal protective equipment (PPE), lack of testing kits, reduced patient volume, workforce furloughs, and lockdowns have forced these units to be innovative and have them prioritize the high-risk procedures and postpone or even cancel endoscopies in medium- to low-risk cases (11).

Outpatient endoscopy centers usually deal with high-volume and close-contact procedures, which could make them prone to become high-risk COVID-19 transmission areas if extreme precautions are not taken. Millions of colonoscopies are performed as part of the colorectal cancer screening program in the United States (US) (12, 13). A wide variety of other therapeutic endoscopic procedures are also performed on a regular basis (14). Furthermore, the staging and palliation of cancers to aid in managing these lesions are increasingly being performed (15). The COVID-19 pandemic has forced these endoscopy centers to drastically reduce the procedure volume for both elective and semi-urgent cases to reduce transmission risk and preserve PPE (16–19).

Furthermore, there are published data about a decrease in the non-variceal GI bleeding events in line with other disorders such as acute coronary syndrome admissions during the pandemic (20). Although the precise reason for these changes remains speculative, patients may have developed a fear of contracting the infection if they visit the medical centers. Additionally, there is an increased risk of exposure to the virus during outpatient endoscopy procedures due to exposure to GI secretions and respiratory secretions (21, 22). Furthermore, there is a potential for increased generation of infected droplets during coughing, retching, and suctioning, creating aerosolization and increased risk of transmission (23, 24).

## Methods

A search for published literature at the time of submission of the manuscript was performed from December 2019 to July 1, 2020. We performed a search of PubMed, Google Scholar, Embase, and Scopus databases to extract articles relevant to endoscopy in COVID-19 patients. The terms “endoscopy,” “gastrointestinal endoscopy,” “staffing,” “barrier protection,” pre-procedure testing,” “COVID-19,” “SARS-CoV-2,” and “coronavirus” were performed. Due to the heterogeneity of the studies, a systemic review could not be performed.

## Changes in the Endoscopy Suites

### Changes in Endoscopy Suite Structure

Various operational changes have been proposed across endoscopy suits/centers to provide services while mitigating the risk of infection. While these changes depend on several factors (availability of resources locally, infection risk, the demographic profile of the patients, indication and hospital/ endoscopy unit policies), common goals of minimizing the risk of transmission of infection, conserving PPE, and achieving high efficiency remain. To achieve these, Cennamo et al. reported substantial changes in the layout of the endoscopy units with risk-based color-coding of the waiting room, endoscopy suites, and recovery room (25). Additionally, implementations of checkpoints, pathways, and processes based on the color-coding schema were implemented in this study (25). Post-procedure, patients are monitored in the recovery area, with no family available in the waiting room. Hospitals in the US have incorporated policies for not allowing family members, given the risk of exposure and transmission (26). Patients are transported to the hospital entrance to find their respective family member/driver who can further assist in discharge. Results of the procedures are discussed with the patient but are relayed via phone to the authorized person with face-to-face encounters (27). While these changes can potentially contribute to the reduction of endoscopy-related transmission, making it safer for the patients and the staff, they also decrease the in-person relay of information, which is critical at the discharge from the endoscopy units.

### Changes in Staffing

COVID-19 first appeared in Wuhan, China, in December 2019. In some areas, the pandemic has overwhelmed the healthcare systems to the point that endoscopy units are potentially treated as COVID-19 units (27, 28). In Brazil, endoscopy staff has been divided into COVID treatment teams and non-COVID endoscopy teams (27). The use of PPE has been mandated by all healthcare systems to minimize the risk of transmission. Prior studies have shown that the use of PPE has not been universal among endoscopists (21, 23). However, with the current pandemic, the use of masks, N-95, gowns, and other PPE has drawn increased attention to avoid spread (24, 29, 30). Endoscopy staff with pre-existing conditions at higher risk of contracting COVID-19 have been assigned non-clinical duties without direct care to COVID-19 patients (27, 28).

Endoscopy staff performing procedures in the operating room should use strict precautions of properly donning and doffing in a separate room prior to entering the operating room. Endoscopy units and operating rooms should follow strict cleaning procedures. Advanced endoscopy procedures such as ERCP frequently need fluoroscopy, equipment trolley, worktable, and anesthesia equipment. The negative pressure rooms are highly recommended in these settings to avoid cross-contamination.

Procedure scheduling during the peak and post-peak has been an area of great challenge for hospitals (31). Increased endoscopy staff furloughs further complicated this challenge (32). Several patient procedures were deferred and put on hold due to decreased slots and uncertainty about when the restrictions will be lifted, and normalcy will be established. Post-procedure telephone follow-ups with patients could be utilized to inquire about developing any new COVID-19 related symptoms to take necessary precautions to individuals who were at potential risk (24, 33). Patients should be informed about the risk of nosocomial infections and also should be informed to report back if they develop any *de novo* symptoms in the next few days after the procedure.

### Change in the Endoscopy Indications

Multiple international societies have recommended restricting endoscopy procedures only to emergent and urgent indications (34). This essential step was taken to minimize the risk of transmission and reduce PPE utilization and use of resources. Studies from multiple countries have shown an endoscopy case-volume median reduction to as high as 99% (19, 35). The impact of COVID-19 varied based on the country, infection rate, initiation of stay-at-home orders, and timing of the pandemic. For example, endoscopy services in the United Kingdom (UK) reduced to 5% in March 2020 after the onset of the pandemic. These changes were noted across all UK regions and endoscopy procedures (36). Procedures were performed only when the benefit outweighs the risk of transmission among the staff.

While the indication for procedures varied, emergent procedures such as active GI bleeding, acute cholangitis, food impactions, and cancer diagnosis/staging/treatment were considered appropriate. A nationwide study in the UK showed that all endoscopic procedures reduced with the pandemic; however, the ERCP activity (performed for emergencies) remains well-preserved (36). Elective procedures, such as screening and surveillance, were deferred. However, the urgent indications of endoscopies remain a gray area based on the endoscopist, institutional guidelines, and available services. Deferring semi-urgent cases could delay the diagnosis of cancers (such as localized pancreatic cancer), and loss of window of therapeutic intervention (endoscopically resectable lesions can become unresectable due to spread). Studies showed that colorectal cancer (CRC) screening declined by 84.5% after the onset of the pandemic in the US (37). Similarly, a 72% reduction in CRC screening was noted in the UK (36). Although multiple GI societies have provided a road map, clinical judgment should prevail, and every case should be individualized with a multidisciplinary team-based approach (38).

### Changes in Triaging

Endoscopy staff triage all patients who are undergoing non-urgent endoscopies. In the US, this triaging is done by the pre-procedural COVID-19 testing and a predetermined questionnaire about 2–3 days before the endoscopy date (39, 40). These patients are again triaged by this questionnaire at the time of presentation to the endoscopy suite. The patients are sent to the hospital to seek emergent medical attention in case they have any signs and symptoms of COVID-19 such as cough, shortness of breath, and persistent fever, along with a known history of contact with a COVID-19 patient or travel to high-risk areas (24, 41). Peri-procedural COVID testing involves coordination at multiple levels—contacting patients to undergo testing 24 to 72 h before the procedure and obtaining the results of the test (42). Patients who travel a long distance to get their procedure may cancel their procedure if their COVID-19 testing results are delayed. Also, the absence of family members before, during, and after the procedure can increase anxiety among the patients.

### Pre-procedural Testing

COVID-19 testing of all patients before endoscopic procedures may help to identify infected patients and facilitate taking appropriate measures such as isolation precautions, high-risk PPE usage for positive PCR testing, and downtime after the procedure. A thorough analysis of the risks and benefits of widespread pre-procedural testing is needed (Table 1). There are some considerations needed before the development and implementation of the universal testing strategy. Testing for all patients incurs cost burden to the endoscopy units, which can be significant. Additionally, testing may delay procedures if test results are pending, and the possibility of false positivity and false negativity might alter decision-making, which can complicate the processes (1, 43).

**Table 1:** Pre-procedural universal testing.

**Advantages**	**Disadvantages**
Results can assist in planning the procedure based on risk and benefit analysis	Significant cost burden
Use of PPE accordingly to negative or positive cases	Risk of false-positives and false-negatives
Planning of the procedure with enhanced precautions and use of minimal personnel (in positive cases) and adequate personnel (in positive cases)	Delay in procedure during to processing times
Decreased transmission risk, reduced downtime and disinfection strategies	Additional trips to the endoscopy center/ testing sites

Corral et al. reported that PCR testing could be used as an effective strategy to restart endoscopic procedures based on the phase of the pandemic (44). Testing individuals within 48 h of the procedure for semi-elective and elective cases can allow completion of 19.4% (if investing $22 per patient) and 95.3% (if investing $105 per patient) of baseline endoscopies. Implementing this strategy over 1 week in the US will return 165 million US dollars (for 13 million investing) and 767 million US dollars (for 64 million investing) (44). These numbers are promising and demonstrate the potential value of COVID-19 testing for all patients undergoing procedures. Expectedly, this modeling can change with the local prevalence of COVID-19, transmission rate (*R*_0_), and accuracy of PCR results. Center for Medicaid and Medicare Services (CMS) and other insurance programs reimburse up to 36 USD to 51 USD per patient (45). These calculations may not apply in areas where testing is not rampant, and reimbursement for endoscopy is low. For example, Sundaram et al. noted that cost of SARS-CoV-2 PCR ($65) might exceed the reimbursement of an upper endoscopy ($30–$60) in countries like India (46). Additionally, the prevalence rate in some of the areas of the country might be too low to test all individuals undergoing endoscopy procedures (46). Testing of high-risk individuals only in specific hot spots is a matter of debate. Furthermore, pre-procedural testing is not uniformly performed in Europe, and the decision is based on the pre-procedure questionnaire. As testing capabilities expand throughout the world, the availability of highly accurate point-of-care testing with rapid results can make this a possibility (47).

### Barrier Protection

Safe distancing in the pre-operative area has decreased the number of patients the nursing staff can receive for pre-operative care. It has affected the efficiency of the endoscopy units severely. Only required and critical personnel (endoscopists, nurses, and anesthetists) should be allowed in the endoscopy units (42). Any instrument or device can potentially be a source of infection in aerosol-generating procedures (AGP). Staff should wear PPE as per the local institutional and national guidelines before starting the procedure. Appropriate donning and doffing of the PPE is essential to reduce the risk of infection (27). Belle et al. noted that gastroenterologists who performed procedures on COVID-19 patients have reported symptoms compatible with COVID-19 ranging from 0.6% (3/497 patients) in low prevalence areas compared to 6.1% (12/197) in high prevalence areas (16). Similarly, Chen et al. reported that 5.7% (8/141 patients) reported that gastroenterologists or their colleagues developed work-related COVID-19 infections (17). It led to the development of multiple barrier devices between endoscopists and patients to reduce the risk of exposure to GI secretions (48–53) (Table 2).

**Table 2:** Barriers to prevent transmission during endoscopy.

**Name**	**Material**	**Description**
Endoprotector (40)	Acrylic plastic	Composed of four faces of the box. Face A (for endoscope insertion), B (for anesthetist), C (air aspiration and creation of negative pressure), D (for patients' neck and shoulders)
C-Cube (42)	Plexiglas	Multiple entryways (endoscopists and anesthesiologists' access) for procedures involving oral cavity
Aerosol box (41)	Plastic	Predominately used for endotracheal intubation. Two circular ports provided for the clinician hands to perform airway procedure
ORIGAMI (43)	Coated cardboard and polypropylene film	Disposable face-protective shield to protect surgical mask and N-95 respiratory mask from aerosols
Endoscopic shield (44)	Plastic cube	Two small holes for endoscopist access to the oral cavity
Chamber unit (45)	Multiple structures	For Ear, Nose, Throat exams- Composed of air inlet, ultraviolent lamps, exhaust system with vents, speaker and additional screen

Given the inherent nature of the procedures (upper or lower endoscopies), endoscopists are almost always in a “high-transmission zone” (within 3 feet of the patients). Campos et al. introduced a transparent aerosol box (endoprotector) to reduce contact with droplets (49). In this technique, a barrier is used during upper endoscopy, which is made of acrylic plastic to shield the respiratory droplets and potential aerosolization during the procedure (49). A similar barrier is used to decrease the exposure to patients' respiratory droplets during endotracheal intubation (ETI) (50). Traina et al. reported the use of an endoscopic COVID Cube (C-Cube), which is a protective box with access to anesthesiologist's hands and another port for endoscope access (51). Liu et al. reported using a unique disposable device with a combined bite block and oxygen mask for upper GI endoscopic procedures (48). Furthermore, a closed chamber ear, nose, and throat (ENT) examination unit was developed for AGP endoscopic examinations of COVID-19 patients (54). While ETI is usually a one-time event to secure the airway, the use of an endoscope through an endoprotector might make the procedure challenging due to the repeated hand movement of the endoscopists. Nevertheless, the use of these barriers has a significant role in reducing the disease transmission, especially in high-risk or COVID-19 patients.

### Endoscopic Transmission

Among the endoscopy procedures, duodenoscopes and echoendoscopes carry a high risk of nosocomial infections (55). While single-use duodenoscopes might be of value in COVID-19-positive patients, they are not universally available and have cost-related constraints (56). Multiple societies have recommended using negative pressure rooms, especially for patients who are suspected of COVID-19 or when the endoscopy is being performed emergently without COVID-19 testing results (30). Intraprocedural changes such as minimal verbal communication, avoiding spill of GI contents via biopsy channel, and avoiding procedures in patients with inadequate bowel preparation should be done (27). Franzini et al. reported the use of a “double gauze technique” where the endoscopists use one gauze and the other by the technician in a controlled fashion to avoid the “whip” effect of accessories and spillage of GI secretions (27). Institutional policies have been developed for minimal personnel to be present for the procedure (57). This is to minimize the risk of exposure among the endoscopy staff. Procedures performed with moderate sedation without the need for anesthesia providers (endoscopist guided sedation) can further minimize the risk of transmission. However, for procedures requiring general anesthesia, societies currently recommend using ETI to reduce the risk of aerosolization with suspected or confirmed COVID-19 (58).

Enhanced cleaning procedures have been implemented by most endoscopy units (24). Strict adherence to local and national policies should be followed while cleaning the endoscopy suites (59). This includes cleaning all horizontal surfaces, frequently touched surfaces with particular emphasis on areas within a few feet of the patient. Multiple studies showed that SARS-CoV-2 can involve any segment of the GI tract. Intestinal autopsy in COVID-19 patients showed stenosis and dilatation of the small intestine (60). Mucosal damage was noted in multiple areas such as esophagus, stomach, duodenum, colon, and rectum (61, 62). Endoscopic procedures, due to their inherent nature of coming in contact with GI secretions, could potentially get contaminated with virus. Although there is a theoretical risk of endoscopes acting as potential vectors for viral infections, so far, there is no published report of SARS-CoV-2 transmission via endoscopes (39). Nevertheless, the reprocessing process should include high-level disinfection (HLD). Traditionally, testing for leakage is performed before washing of the endoscope. However, suggestions have been made for performing this after washing the endoscope. Whether this can affect the proper functioning of the scope remains to be studied (48).

### Procedural Downtime

Patients undergoing endoscopy have the potential risk of aerosol generation. All rooms after the procedure should be deemed contaminated after the procedure. During the induction of anesthesia, only essential personnel for securing the airway should be present, which requires endoscopy staff to wait outside the procedure room. After completing the endoscopic procedure, the endoscopist and non-essential staff should exit the room before extubating the patient.

The time needed to allow for dispersion of the virus-laden aerosols to clear will depend on the rate of air changes/hour (ACH). If a rate of 25–30 cycles/hour is used, 3 min are needed to wait before a procedure could be started after intubation. The precise time needed for closure of the room depends on the use of negative pressure and air-exchange rate (63). While this is dependent on transmission dynamics, multiple other factors such as air-exchange rate, duration of aerosolized droplets suspended in the air, viral load in the droplets, the viability of the virus (can be up to 3 h), and environmental contamination could play a role (Table 3).

**Table 3:** Factors^*^ predicting downtime between endoscopic procedures.

**Increased downtime (increased delay between procedures)**	**Decreased downtime (decreased delay between procedures)**
High viral load in the droplet secretions (contaminant concentration)	High air changes per hour (ACH)
Heavy environmental contamination	Efficient vent system (removal efficiency)
Air stagnation	Negative pressure room availability
Large room volume	Good mixing of the air within the space

* *Final factors determining the downtime is dependent on transmission dynamics, manufacturer recommendations and contaminant concentrations*.

A cautious approach is recommended until further data emerge (19). Per the CDC (63), airborne contaminant removal is dependent on ACH and duration, which determines the efficiency of removal. For example, a room with a minimum ACH of 12–15 cycles per hour, at a duration of 28 min to 35 min, is needed to achieve a contaminant removal efficiency of 99.9% (63). This efficiency comes down if the ACH is low and if viral contamination in the air is high. Societies have recommended adequately ventilated rooms (with at least 12 ACH and controlled direction of airflow with mechanical ventilation should be used). For rooms without negative pressure capability, a minimum of 60 min delay (downtime) is recommended compared to 30-min downtime for negative pressure room (24).

### Endoscopy Trainee Involvement

Endoscopy trainees' (gastroenterology fellows and surgery residents) involvement in GI procedures have been affected significantly during the pandemic (64, 65). A multinational survey study spanning 63 countries reported a reduction of endoscopy volume by up to 93.8%, with colonoscopy being affected the most, which is a core endoscopy skill (35). Furthermore, an increased degree of anxiety and burnout were noted among endoscopists. This concern not only was restricted to trainees but also affected entire endoscopy staff for the risk of acquiring COVID-19, especially after the resumption of elective endoscopies (66). However, in areas with low risk of infection transmission, trainees continue to be involved in the procedures.

During the initial phase of the pandemic, most endoscopy units implemented policies to have only essential fully trained staff to avoid exposure and reduce the turnover time (30). Multiple survey studies have demonstrated adverse effects on endoscopy training and an unexpectedly significant fear and anxiety during pandemic (35, 67, 68). Multiple GI and surgical societies have increased the availability of electronic resources to fill this gap in the training (69, 70). Additionally, programs have implemented various mechanisms to mitigate the loss of procedure volume with video recording, simulation labs, and increasing involvement in low-risk procedures.

## Future Outlook of Endoscopy Units

In the future, endoscopy units will likely incorporate some of the changes during the pandemic for increased safety of the patients and endoscopy staff. It remains speculative to predict the end of this pandemic, but localized outbreaks may continue to occur even when we see a pandemic downtrend (71). Important questions remain open if endoscopy staff and patients should continue to be screened and tested regularly. It is only presumptive to say about the effect of cancer burden due to delayed screening, surveillance, and handling the increased backlog cases. Multiple strategies can be adopted to decrease or ease endoscopy demand. Patients who are eligible for the screening should be provided with options of CRC screening, including stool-based testing, which do not need patients to present to the healthcare facilities. Home-based stool testing has the advantage of testing without contact with hospitals or clinics (72). For patients who test positive, there is a significant risk of advanced adenomas on the endoscopy, and hence a triage system should be developed to prioritize the procedures (72). Because of these, there will be increased demand in the recovery phase that likely needs to be phased appropriately to avoid significant waiting times for procedures that need to happen in a timely fashion. It involves careful evaluation of patient demographics (comorbidities) and environmental factors (staff availability, local resources, community spread, and infection rate) (73). As the recovery phase starts, the real effect on the delay in cancer screening will emerge.

Patients should be communicated about the importance of screenings in the recovery phase to avoid delays and to keep the appointments. There should be an effective use of electronic health record communication strategies to provide updates to patients about COVID-19-related changes in endoscopy units. Virtual tools such as increased telehealth visits to discuss and engage patients about cancer screening programs will increase the endoscopy show rates (74). A triage system to review all the posted case by qualified medical personnel and reschedule the procedures in a tiered fashion can make this process less stressful (42). Furthermore, endoscopy staff should communicate with schedulers about the patient's concerns, which can be directly addressed. Finally, a higher threshold should be adopted for endoscopy procedures, which will less likely change the outcomes in patients (75). Despite these changes, as this pandemic unfolds with localized outbreaks, endoscopy units remain at a threat of temporary closures and need for enhanced disinfection protocols. Preparing for future pandemics should be a part of the operation of the endoscopy units' stress response. Nevertheless, endoscopy units should continue to adapt and navigate to provide high-quality patient care with equal emphasis on patient and staff safety.

## Limitations

Due to the rapidly evolving nature of the COVID-19 pandemic, endoscopy units continue to adapt, and the above recommendation can change. Due to the heterogeneity of the published literature, we could not perform a systematic review. Pre-procedural testing, triaging, and trainee involvement in the procedures are dependent on infection risk, local endoscopy unit, and hospital policies. As countries are starting the recovery phase of the COVID-19 pandemic, these measures are constantly being updated.

## Conclusion

Endoscopy units are on the verge of significant changes and evolution with the unfolding of the COVID-19 pandemic. The current pandemic calls for multiple changes at different levels not only to perform procedures in a safe environment for patients but also to prevent infection to the endoscopy staff. It appears likely that COVID-19 will be an integral part of our lives, like other viruses such as influenza. Similar to other procedures' predominant specialties, endoscopy units are incorporating operational changes in order to provide care in these unprecedented times. The use of enhanced protocols with particular emphasis on assessing the risk status of the patient, proper use of PPE, and perioperative procedural changes incorporated during this pandemic should be a lesson for the future. While some of these changes can gain permanent stance in the future, adapting to the future outbreaks is critical to provide excellent care.

## Author Contributions

HG and AP: conception and design and literature review. AP: first draft. All authors: critical revision, editing, and final approval. All authors contributed to the article and approved the submitted version.

## Conflict of Interest

The authors declare that the research was conducted in the absence of any commercial or financial relationships that could be construed as a potential conflict of interest.
